# 1,4-Bis(hex­yloxy)-2,5-diiodo­benzene

**DOI:** 10.1107/S1600536810005258

**Published:** 2010-03-13

**Authors:** Damien Thevenet, Reinhard Neier, Olha Sereda, Antonia Neels, Helen Stoeckli-Evans

**Affiliations:** a Institute of Chemistry, University of Neuchâtel, rue Emile-Argand 11, 2009 Neuchâtel, Switzerland; b XRD Application LAB, Microsystems Technology Division, Swiss Center for Electronics and Microtechnology, rue Jaquet Droz 1, CH-2001 Neuchâtel, Switzerland; c Institute of Physics, University of Neuchâtel, rue Emile-Argand 11, 2009 Neuchâtel, Switzerland

## Abstract

The centrosymmetric title compound, C_18_H_28_I_2_O_2_, crystallized in the monoclinic space group *P*2_1_/*c* with the alkyl chains having extended all-*trans* conformations, similar to those in the centrosymmetric bromo analogue [Li *et al.* (2008 ▶). *Acta Cryst.* E**64**, o1930] that crystallized in the triclinic space group *P* 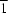. The difference between the two structures lies in the orientation of the two alkyl chains with respect to the C(aromatic)—O bond. In the title compound, the *O*—C_alk­yl_—C_alk­yl_—C_alk­yl_ torsion angle is 55.8 (5)°, while in the bromo analogue this angle is −179.1 (2)°. In the title compound, the C-atoms of the alkyl chain are almost coplanar [maximum deviation of 0.052 (5) Å] and this mean plane is inclined to the benzene ring by 50.3 (3)°. In the bromo-analogue, these two mean planes are almost coplanar, making a dihedral angle of 4.1 (2)°. Another difference between the crystal structures of the two compounds is that in the title compound there are no halide⋯halide inter­actions. Instead, symmetry-related mol­ecules are linked *via* C—H⋯π contacts, forming a two-dimensional network.

## Related literature

For use of the title compound in the synthesis of conjugated polymers, see: Van Heyningen *et al.* (2003 ▶); Mayor & Didschies (2003 ▶). For the various syntheses of the title compound, see: Castanet *et al.* (2002 ▶); Van Heyningen *et al.* (2003 ▶); Mayor & Didschies (2003 ▶); Plater *et al.* (2004 ▶). For the synthesis and crystal structure of the bromo analogue, see: Maruyama & Kawanishi (2002 ▶); Li *et al.* (2008 ▶). For bond distances, see Allen *et al.* (1987 ▶).
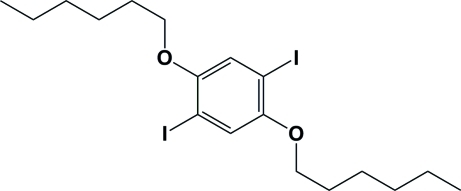


## Experimental

### 

#### Crystal data

C_18_H_28_I_2_O_2_
*M* *_r_* = 530.20
Monoclinic, 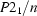

*a* = 9.4481 (9) Å
*b* = 7.8455 (6) Å
*c* = 13.457 (2) Å
β = 92.148 (12)°
*V* = 996.80 (16) Å^3^
*Z* = 2
Mo *K*α radiation
μ = 3.16 mm^−1^
*T* = 173 K
0.32 × 0.11 × 0.06 mm

#### Data collection

STOE IPDS diffractometer
Absorption correction: multi-scan *MULscanABS* in *PLATON* (Spek, 2009 ▶) *T*_min_ = 0.952, *T*_max_ = 1.042
7660 measured reflections
1962 independent reflections
1216 reflections with *I* > 2σ(*I*)
*R*_int_ = 0.058

#### Refinement

*R*[*F* ^2^ > 2σ(*F* ^2^)] = 0.029
*wR*(*F* ^2^) = 0.055
*S* = 0.79
1962 reflections
101 parameters
H-atom parameters constrained
Δρ_max_ = 0.81 e Å^−3^
Δρ_min_ = −1.31 e Å^−3^

### 

Data collection: *EXPOSE* in *IPDS-I* (Stoe & Cie, 2000 ▶); cell refinement: *CELL* in *IPDS-I*; data reduction: *INTEGRATE* in *IPDS-I*; program(s) used to solve structure: *SHELXS97* (Sheldrick, 2008 ▶); program(s) used to refine structure: *SHELXL97* (Sheldrick, 2008 ▶); molecular graphics: *Mercury* (Macrae *et al.*, 2006 ▶); software used to prepare material for publication: *SHELXL97* and *PLATON* (Spek, 2009 ▶).

## Supplementary Material

Crystal structure: contains datablocks I, global. DOI: 10.1107/S1600536810005258/lx2134sup1.cif

Structure factors: contains datablocks I. DOI: 10.1107/S1600536810005258/lx2134Isup2.hkl

Additional supplementary materials:  crystallographic information; 3D view; checkCIF report

## Figures and Tables

**Table 1:** C—H⋯π inter­actions (Å, °) *Cg*1 is the centroid of the C1–C3/C1^i^–C3^i^ ring.

D—H⋯centroid	C—H	H⋯*Cg*	D⋯*Cg*	C—H⋯*Cg*
C4′—H4′2⋯*Cg*^ii^	0.99	2.74	3.595 (5)	145.0

Symmetry codes: (i) 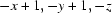; (ii) 
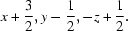

**Fig. 1.**
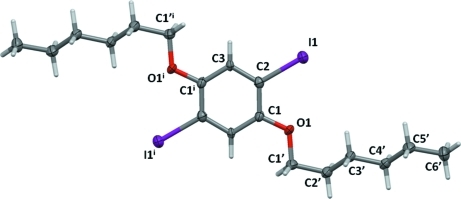
A view of the molecular structure of the title compound, with displacement ellipoids drawn at the 50% probabilty level. Atoms labelled i are related to the other atoms by the symmetry operation -x+1, -y+1, -z.

**Fig. 2.**
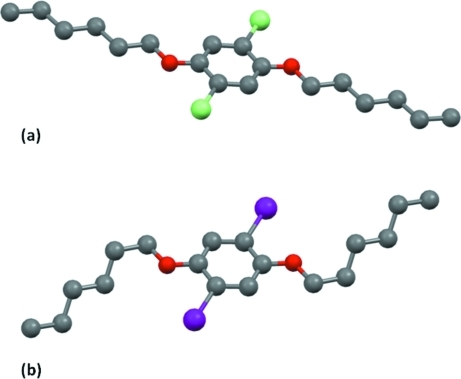
A view of the different molecular conformations in (a) the bromo-analogue (Li et al., 2008), and (b) the title compound. The H-atoms have been omitted for clarity.

**Fig. 3.**
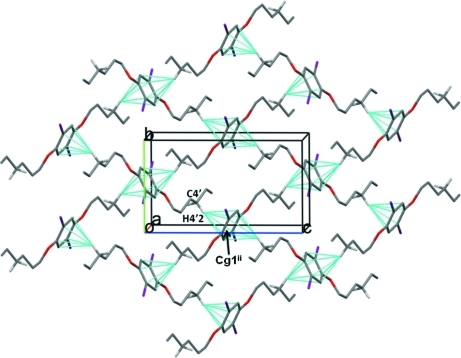
A view along the a-axis of the crystal packing in the title compound. The C—H···π interactions are illustrated by the H···C contacts [H4'2···C-atoms of the benzene ring] of 2.9–3.2 Å, drawn as dotted cyan lines. H-atoms not involved in the C—H···π interactions have been omitted for clarity; symmetry code (ii) -x+3/2, y-1/2, -z+1/2.

## supplementary crystallographic information

### Comment

The title compound has been used as a building block for the elaboration of organic–electronic materials, for example as a monomer for the synthesis of conjugated polymers (Van Heyningen *et al.*, 2003; Mayor & Didschies, 2003). Our interest in this compound lies in the possibility of using it as a spacer-unit in linked materials for the creation of structured, discotic mesophases. The synthesis of the title compound has been reported by various groups (Van Heyningen *et al.*, 2003; Mayor & Didschies, 2003; Plater *et al.*, 2004). Here it was synthesized by iodination of 1,4-bis(hexyloxy)benzene (Castanet *et al.*, 2002). The crystal structure of the bromo-analogue, synthesized by (Maruyama & Kawanishi, 2002), has been described by (Li *et al.*, 2008).

The molecular structure of the title compound is illustrated in Fig. 1. Bond lengths are normal (Allen *et al.*, 1987) and similar to those in the bromo-analogue (Li *et al.*,  2008). The molecule possesses C_i_ symmetry with the inversion center situated at the center of the aromatic ring. The alkyl chains adopt a fully extended all-*trans* conformation. The C-atoms of the alkyl chain are almost coplanar (max. deviation of 0.052 (5) Å) and this mean plane is inclined to the benzene ring by 50.3 (3)°. In the bromo-analogue the alkyl chains also adopt a fully extended all-*trans* conformation. The alkyl C-atoms are also coplanar [max. deviation of 0.034 (4) Å] but here lie almost in the same plane as the aromatic ring, with a dihedral angle of 4.1 (2)°.

The different comformations of the two compounds are illustrated in Fig. 2. It can be seen that the alkyl chains are orientated differently with respect to the C(aromatic)—O bonds. The O1—C1'—C2'—C3' torsion angle is 55.8 (5)° in the title compound (Fig. 2b), while in the bromo-analogue this same angle is -179.1 (2)° (Fig. 2a). In the crystal structure of the title compound there are no halide···halide interactions, in contrast to the Br···Br interactions [3.410 (3) Å] observed in the bromo-analogue. However, symmetry related molecules are linked by C—H···π interactions leading to the formation of a two-dimensional network (Table 1 and Fig. 3; Cg is the centroid of the C1–C3/C1^i^–C3^i^ benzene ring).

### Experimental

The title compound was synthesized by iodination of 1,4-bis(hexyloxy)benzene (Castanet *et al.*, 2002). To a solution of 1,4-bis(hexyloxy)benzene (0.75 mmol) and *N*-iodosuccinimide (2.40 mmol) in dry acetonitrile (5.0 ml) was added trifluoroacetic acid (1.50 mmol) at RT. The mixture was heated and stirred at 363 K for 2 h. The reaction mixture was then cooled to RT and concentrated. Diethyl ether (30 ml) was added and the heterogeneous mixture was filtered to remove the white precipitate of succinimide that had formed. The organic layer was then washed with 10% NaHSO_3_ (aq) (3 × 30 ml) and dried over MgSO_4_. The crude product was purified by column chromatography [silica gel, Petroleum ether : CH_2_Cl_2_ (5:1)] and recrystallisation in methanol. Single crystals of the title compound were grown by slow evaporation of a concentrated solution in CH_2_Cl_2_ at RT. ^1^H NMR, 400 MHz (CDCl_3_) δ 7.17 (s, 2H, H_3,3^i^_), 3.93 (t, J = 6.6 Hz, 4H, H_1'_), 1.80 (quint, J = 6.6 Hz, 4H, H_2'_), 1.50 (m, 4H, H_3'_), 1.35 (m, 8H, H_4',5'_), 0.91 (t, J = 7.0 Hz, H_6'_); ^13^C NMR, 100 MHz (CDCl_3_) δ 152.8 (C_2,2^i^_), 122.7 (C_3,3^i^_), 86.3 (C_1,1^i^_), 70.3 (C_1'_), 31.4 (C_5'_), 29.1 (C_2'_), 25.7 (C_3'_), 22.6 (C_4'_), 14.0 (C_6'_); MS (EI): [M]^+^ = 529.95. The same numbering scheme has been used for the crystal structure.

### Refinement

The H-atoms could all be located in difference electron-density maps. In the final cycles of refinement they were included in calculated positions and treated as riding atoms: C—H = 0.98 - 0.99 Å, with *U*_iso_(H) = k × *U*_eq_(parent C-atom), where k = 1.2 for H-aromatic and H-methylene, and 1.5 for H-methyl.

### Crystal data

**Table:**

C_18_H_28_I_2_O_2_	*F*(000) = 516
*M**_r_* = 530.20	*D*_x_ = 1.767 Mg m^−^^3^
Monoclinic, *P*2_1_/*n*	Mo *K*α radiation, λ = 0.71073 Å
Hall symbol: -P 2yn	Cell parameters from 7553 reflections
*a* = 9.4481 (9) Å	θ = 0.9–26.3°
*b* = 7.8455 (6) Å	µ = 3.16 mm^−^^1^
*c* = 13.457 (2) Å	*T* = 173 K
β = 92.148 (12)°	Rod, colorless
*V* = 996.80 (16) Å^3^	0.32 × 0.11 × 0.06 mm
*Z* = 2	

### Data collection

**Table:**

STOE IPDS diffractometer	1962 independent reflections
Radiation source: fine-focus sealed tube	1216 reflections with *I* > 2σ(*I*)
graphite	*R*_int_ = 0.058
φ rotation scans	θ_max_ = 26.1°, θ_min_ = 2.6°
Absorption correction: multi-scan MULscanABS in *PLATON* (Spek, 2009)	*h* = −11→11
*T*_min_ = 0.952, *T*_max_ = 1.042	*k* = −9→9
7660 measured reflections	*l* = −16→16

### Refinement

**Table:**

Refinement on *F*^2^	Primary atom site location: structure-invariant direct methods
Least-squares matrix: full	Secondary atom site location: difference Fourier map
*R*[*F*^2^ > 2σ(*F*^2^)] = 0.029	Hydrogen site location: difference Fourier map
*wR*(*F*^2^) = 0.055	H-atom parameters constrained
*S* = 0.79	*w* = 1/[σ^2^(*F*_o_^2^) + (0.0227*P*)^2^] where *P* = (*F*_o_^2^ + 2*F*_c_^2^)/3
1962 reflections	(Δ/σ)_max_ < 0.001
101 parameters	Δρ_max_ = 0.81 e Å^−^^3^
0 restraints	Δρ_min_ = −1.31 e Å^−^^3^

### Special details

**Table:**

Geometry. Bond distances, angles etc. have been calculated using the rounded fractional coordinates. All su's are estimated from the variances of the (full) variance-covariance matrix. The cell esds are taken into account in the estimation of distances, angles and torsion angles
Refinement. Refinement of *F*^2^ against ALL reflections. The weighted *R*-factor wR and goodness of fit *S* are based on *F*^2^, conventional *R*-factors *R* are based on *F*, with *F* set to zero for negative *F*^2^. The threshold expression of *F*^2^ > σ(*F*^2^) is used only for calculating *R*-factors(gt) etc. and is not relevant to the choice of reflections for refinement. *R*-factors based on *F*^2^ are statistically about twice as large as those based on *F*, and *R*- factors based on ALL data will be even larger.

### Fractional atomic coordinates and isotropic or equivalent isotropic displacement parameters (Å^2^)

**Table:**

	*x*	*y*	*z*	*U*_iso_*/*U*_eq_	
I1	0.82998 (3)	0.69287 (4)	0.01561 (3)	0.0307 (1)	
O1	0.7295 (3)	0.3497 (4)	0.0975 (2)	0.0276 (10)	
C1	0.6122 (4)	0.4192 (6)	0.0508 (3)	0.0215 (14)	
C1'	0.7158 (4)	0.1961 (7)	0.1533 (3)	0.0279 (16)	
C2	0.6307 (4)	0.5768 (6)	0.0054 (3)	0.0214 (16)	
C2'	0.8638 (5)	0.1475 (6)	0.1902 (4)	0.0286 (16)	
C3	0.5180 (4)	0.6593 (6)	−0.0459 (3)	0.0170 (14)	
C3'	0.9402 (4)	0.2857 (6)	0.2498 (3)	0.0242 (16)	
C4'	1.0897 (5)	0.2350 (6)	0.2818 (3)	0.0261 (16)	
C5'	1.1699 (5)	0.3737 (6)	0.3398 (4)	0.0332 (17)	
C6'	1.3227 (5)	0.3225 (8)	0.3657 (4)	0.0373 (16)	
H1'1	0.67420	0.10460	0.11080	0.0340*	
H1'2	0.65390	0.21470	0.21010	0.0340*	
H3	0.53150	0.76690	−0.07650	0.0210*	
H2'1	0.92060	0.11840	0.13220	0.0340*	
H2'2	0.85780	0.04420	0.23210	0.0340*	
H3'1	0.94350	0.39070	0.20920	0.0290*	
H3'2	0.88620	0.31150	0.30960	0.0290*	
H4'1	1.14270	0.20610	0.22190	0.0310*	
H4'2	1.08590	0.13140	0.32350	0.0310*	
H5'1	1.12050	0.39790	0.40180	0.0400*	
H5'2	1.16960	0.47950	0.29970	0.0400*	
H6'1	1.32350	0.21600	0.40380	0.0560*	
H6'2	1.36880	0.41260	0.40570	0.0560*	
H6'3	1.37380	0.30610	0.30440	0.0560*	

### Atomic displacement parameters (Å^2^)

**Table:**

	*U*^11^	*U*^22^	*U*^33^	*U*^12^	*U*^13^	*U*^23^
I1	0.0231 (1)	0.0311 (2)	0.0374 (2)	−0.0054 (2)	−0.0051 (1)	0.0055 (2)
O1	0.0202 (14)	0.026 (2)	0.0358 (19)	−0.0003 (12)	−0.0093 (13)	0.0145 (15)
C1	0.019 (2)	0.021 (3)	0.024 (2)	0.0025 (18)	−0.0054 (18)	−0.001 (2)
C1'	0.028 (2)	0.021 (3)	0.034 (3)	−0.003 (2)	−0.0063 (19)	0.007 (3)
C2	0.021 (2)	0.023 (3)	0.020 (3)	−0.0020 (18)	−0.0013 (17)	−0.003 (2)
C2'	0.029 (2)	0.025 (3)	0.031 (3)	0.001 (2)	−0.008 (2)	0.005 (2)
C3	0.0108 (19)	0.022 (3)	0.018 (2)	−0.0001 (18)	−0.0003 (16)	−0.001 (2)
C3'	0.022 (2)	0.023 (3)	0.027 (3)	0.002 (2)	−0.0054 (18)	0.004 (2)
C4'	0.028 (2)	0.026 (3)	0.024 (3)	0.0020 (18)	−0.004 (2)	0.006 (2)
C5'	0.031 (3)	0.025 (3)	0.043 (3)	−0.001 (2)	−0.007 (2)	0.008 (2)
C6'	0.027 (2)	0.039 (3)	0.045 (3)	−0.006 (3)	−0.010 (2)	0.002 (3)

### Geometric parameters (Å, °)

**Table:**

I1—C2	2.091 (4)	C2'—H2'1	0.9900
O1—C1	1.367 (5)	C2'—H2'2	0.9900
O1—C1'	1.428 (6)	C3—H3	0.9500
C1—C2	1.393 (6)	C3'—H3'1	0.9900
C1—C3^i^	1.375 (6)	C3'—H3'2	0.9900
C1'—C2'	1.515 (6)	C4'—H4'1	0.9900
C2—C3	1.405 (6)	C4'—H4'2	0.9900
C2'—C3'	1.515 (7)	C5'—H5'1	0.9900
C3'—C4'	1.514 (6)	C5'—H5'2	0.9900
C4'—C5'	1.524 (7)	C6'—H6'1	0.9800
C5'—C6'	1.526 (7)	C6'—H6'2	0.9800
C1'—H1'1	0.9900	C6'—H6'3	0.9800
C1'—H1'2	0.9900		
			
I1···O1	3.073 (3)	H2'1···H4'1	2.4800
I1···C6'^ii^	3.736 (5)	H2'2···H4'2	2.5400
I1···H3'2^iii^	3.3100	H2'2···O1^vii^	2.9000
I1···H4'1^iv^	3.3100	H2'2···C1^vii^	3.0800
O1···I1	3.073 (3)	H3'1···O1	2.4900
O1···H3'1	2.4900	H3'1···H5'2	2.5200
O1···H2'2^iii^	2.9000	H3'2···H5'1	2.5900
O1···H6'1^v^	2.8300	H3'2···I1^vii^	3.3100
C6'···I1^vi^	3.736 (5)	H4'1···H2'1	2.4800
C1···H4'2^iii^	3.0600	H4'1···H6'3	2.5400
C1···H4'2^v^	3.0900	H4'1···H5'2^vi^	2.5400
C1···H2'2^iii^	3.0800	H4'1···I1^iv^	3.3100
C1···H6'1^v^	3.0500	H4'2···H2'2	2.5400
C1'···H3^i^	2.5400	H4'2···H6'1	2.5400
C2···H4'2^v^	2.9600	H4'2···C1^vii^	3.0600
C3···H5'1^iii^	3.0300	H4'2···C1^viii^	3.0900
C3···H1'2^i^	2.8700	H4'2···C2^viii^	2.9600
C3···H4'2^v^	2.9600	H4'2···C3^viii^	2.9600
C3···H1'1^i^	2.7200	H5'1···H3'2	2.5900
H1'1···C3^i^	2.7200	H5'1···C3^vii^	3.0300
H1'1···H3^i^	2.2200	H5'2···H3'1	2.5200
H1'2···C3^i^	2.8700	H5'2···H4'1^ii^	2.5400
H1'2···H3^i^	2.4700	H6'1···H4'2	2.5400
H3···C1'^i^	2.5400	H6'1···O1^viii^	2.8300
H3···H1'1^i^	2.2200	H6'1···C1^viii^	3.0500
H3···H1'2^i^	2.4700	H6'3···H4'1	2.5400
			
C1—O1—C1'	119.4 (3)	C2—C3—H3	121.00
O1—C1—C2	116.3 (3)	C1^i^—C3—H3	121.00
O1—C1—C3^i^	123.5 (4)	C2'—C3'—H3'1	109.00
C2—C1—C3^i^	120.2 (4)	C2'—C3'—H3'2	109.00
O1—C1'—C2'	106.5 (3)	C4'—C3'—H3'1	109.00
I1—C2—C1	119.0 (3)	C4'—C3'—H3'2	109.00
I1—C2—C3	119.7 (3)	H3'1—C3'—H3'2	108.00
C1—C2—C3	121.3 (4)	C3'—C4'—H4'1	109.00
C1'—C2'—C3'	114.1 (4)	C3'—C4'—H4'2	109.00
C1^i^—C3—C2	118.5 (4)	C5'—C4'—H4'1	109.00
C2'—C3'—C4'	112.5 (4)	C5'—C4'—H4'2	109.00
C3'—C4'—C5'	113.5 (4)	H4'1—C4'—H4'2	108.00
C4'—C5'—C6'	112.1 (4)	C4'—C5'—H5'1	109.00
O1—C1'—H1'1	110.00	C4'—C5'—H5'2	109.00
O1—C1'—H1'2	110.00	C6'—C5'—H5'1	109.00
C2'—C1'—H1'1	110.00	C6'—C5'—H5'2	109.00
C2'—C1'—H1'2	110.00	H5'1—C5'—H5'2	108.00
H1'1—C1'—H1'2	109.00	C5'—C6'—H6'1	109.00
C1'—C2'—H2'1	109.00	C5'—C6'—H6'2	109.00
C1'—C2'—H2'2	109.00	C5'—C6'—H6'3	110.00
C3'—C2'—H2'1	109.00	H6'1—C6'—H6'2	109.00
C3'—C2'—H2'2	109.00	H6'1—C6'—H6'3	110.00
H2'1—C2'—H2'2	108.00	H6'2—C6'—H6'3	110.00
			
C1'—O1—C1—C2	174.3 (4)	C2—C1—C3^i^—C2^i^	−0.2 (6)
C1'—O1—C1—C3^i^	−6.7 (6)	O1—C1'—C2'—C3'	55.8 (5)
C1—O1—C1'—C2'	176.8 (4)	I1—C2—C3—C1^i^	−179.5 (3)
O1—C1—C2—I1	−1.5 (5)	C1—C2—C3—C1^i^	−0.2 (6)
O1—C1—C2—C3	179.2 (4)	C1'—C2'—C3'—C4'	−177.7 (4)
C3^i^—C1—C2—I1	179.5 (3)	C2'—C3'—C4'—C5'	178.6 (4)
C3^i^—C1—C2—C3	0.2 (6)	C3'—C4'—C5'—C6'	−176.7 (4)
O1—C1—C3^i^—C2^i^	−179.1 (4)		

Symmetry codes: (i) −*x*+1, −*y*+1, −*z*; (ii) −*x*+5/2, *y*+1/2, −*z*+1/2; (iii) −*x*+3/2, *y*+1/2, −*z*+1/2; (iv) −*x*+2, −*y*+1, −*z*; (v) *x*−1/2, −*y*+1/2, *z*−1/2; (vi) −*x*+5/2, *y*−1/2, −*z*+1/2; (vii) −*x*+3/2, *y*−1/2, −*z*+1/2; (viii) *x*+1/2, −*y*+1/2, *z*+1/2.

### Table 1 C—H···π interactions (Å, °)

Cg1 is the centroid of the C1–C3/C1^i^–C3^i^ ring.

**Table:**

D—H···centroid	C—H	H···Cg	D···Cg	C—H···Cg
C4'—H4'2···Cg^ii^	0.99	2.74	3.595 (5)	145.0

Symmetry codes: (i) -x+1, -y+1, -z; (ii) -x+3/2, y-1/2, -z+1/2.
